# The Molecular Network of Neutrophil Extracellular Traps in Hepatocellular Carcinoma: Biogenesis, Function, and Therapeutic Implications

**DOI:** 10.3390/molecules31040749

**Published:** 2026-02-23

**Authors:** Chang Liu, Jienan Lu, Yang Tian, Sinan Lu, Weili Wang, Jun Jiang, Xiang Zheng, Sheng Yan

**Affiliations:** 1 Department of Hepatobiliary and Pancreatic Surgery, The Second Affiliated Hospital, Zhejiang University School of Medicine, Hangzhou 310009, China; liuchang42398@163.com (C.L.); 21118058@zju.edu.cn (Y.T.); lusinan@163.cm (S.L.); wangweili@zju.edu.cn (W.W.); 2 Zhejiang University School of Medicine, Hangzhou 310058, China; 3 Department of Nursing, The Second Affiliated Hospital, Zhejiang University School of Medicine, Hangzhou 310009, China; 2504051@zju.edu.cn

**Keywords:** neutrophil extracellular traps, NETosis, hepatocellular carcinoma, tumor microenvironment

## Abstract

Hepatocellular carcinoma (HCC) remains the leading cause of cancer-related death worldwide. Its high aggressiveness and resistance to therapy arise, in large part, from an immunosuppressive tumor microenvironment (TME). Neutrophil extracellular traps (NETs) are web-like assemblies of chromatin and granular proteins released during NETosis, and they have emerged as major inflammatory drivers within the HCC TME. NETs actively promote tumor progression by physically trapping circulating tumor cells, remodeling the extracellular matrix, stimulating angiogenesis, and facilitating immune evasion. In this review, we systematically dissect the molecular networks that link NETs to HCC. We summarize the signaling pathways that regulate NETs formation, detail the multifaceted roles of NETs in hepatocarcinogenesis, metastasis, and therapy resistance, and assess the translational potential of NETs as diagnostic biomarkers and therapeutic targets. Together, these analyses offer theoretical guidance for developing the next generation of precision-medicine strategies for HCC.

## 1. Introduction

Global statistics for 2022 reported about 865,000 new cases of liver cancer and 758,000 deaths; liver cancer therefore remains a leading cause of cancer-related mortality worldwide [1]. Hepatocellular carcinoma (HCC) is the dominant histological subtype, comprising 75–85% of primary liver cancers [2]. Patients with early-stage HCC can achieve favorable outcomes after local treatments such as surgical resection [3]. However, most patients present with advanced disease when therapeutic options are limited. Those with advanced HCC frequently develop acquired resistance to systemic agents—including tyrosine kinase inhibitors (e.g., sorafenib and lenvatinib) and immune checkpoint inhibitors (ICIs)—resulting in disease progression and markedly reduced overall survival [4,5]. A growing body of evidence links HCC’s aggressive biology and treatment resistance to a highly immunosuppressive, chronically proinflammatory tumor microenvironment (TME) [6]. Neutrophils are among the first innate immune cells to infiltrate tumor tissue, and higher neutrophil infiltration correlates with poorer patient prognosis [7]. Tumor-associated neutrophils (TANs) display marked phenotypic heterogeneity and act as double-edged regulators in HCC pathogenesis through complex cytokine signaling networks; however, the molecular mechanisms that govern this functional switching remain incompletely defined [8,9]. Thus, a detailed analysis of neutrophil regulatory networks in HCC carries both significant scientific interest and translational potential.

In recent years, researchers have identified a previously unrecognized functional program in neutrophils: the formation of neutrophil extracellular traps (NETs) [10]. NETs are extracellular, web-like scaffold structures composed primarily of decondensed chromatin decorated with granule-derived proteins, including neutrophil elastase (NE) and myeloperoxidase (MPO) [10]. Neutrophils release these structures through a specialized form of cell death known as NETosis [11]. Within the TME, dysregulated NET formation provides both a physical scaffold and a biochemical platform that drives malignant progression, metastasis, and tumor recurrence. Mechanistically, NETs promote these pathological processes by trapping circulating tumor cells, remodeling the extracellular matrix, and facilitating immune escape [12,13,14].

Accumulating evidence demonstrates that NETs are widely distributed throughout the HCC microenvironment, and their abundance strongly correlates with disease progression, metastasis, and poor clinical outcomes [15,16]. Recent studies have begun to define the specific molecular signaling axes that underlie these effects. CD^8+^ T-cells serve as the primary effectors of anti-tumor immunity in the HCC microenvironment; however, their functional state is often compromised by the suppressive niche, leading to a state of exhaustion characterized by the high expression of inhibitory receptors and diminished cytolytic capacity [17]. NET-derived DNA impairs antitumor immunity by binding to the transmembrane and coiled-coil domains 6 (TMCO6) receptor on CD^8+^ T-cells [18]. In the setting of non-alcoholic steatohepatitis (NASH), NETs further promote immunosuppression by enhancing the recruitment of regulatory T-cells (Tregs) [19]. Moreover, the HCC microenvironment induces the release of NETs enriched with oxidized mitochondrial DNA (ox-mtDNA), which exhibit strong proinflammatory and prometastatic activity [20]. Together, these findings underscore the significant translational potential of NETs as diagnostic biomarkers and therapeutic targets in HCC.

The clinical translation of NETs-targeted therapies remains limited by several major challenges. First, our understanding of the NETs regulatory network is incomplete. We lack a comprehensive hierarchical framework that connects upstream triggers in specific pathological contexts to activation of the core NETosis machinery and downstream intracellular signaling pathways [21]. Second, accurate quantification of NETs remains technically constrained. No gold standard method exists, and current approaches rely on surrogate markers, such as citrullinated histone H3 (CitH3) and MPO–DNA complexes. These markers lack specificity and do not capture the heterogeneity of NETs subtypes [22,23,24]. Third, NETs exhibit functional duality. Although they predominantly promote HCC progression, they can exert cytotoxic or T-cell-recruiting antitumor effects in other cancers, including bladder cancer and melanoma [25,26]. In addition, trained immunity may reprogram neutrophils toward an antitumor phenotype [27]. Therefore, we must identify the key determinants that shift this functional balance within the HCC microenvironment.

**Figure 1:**
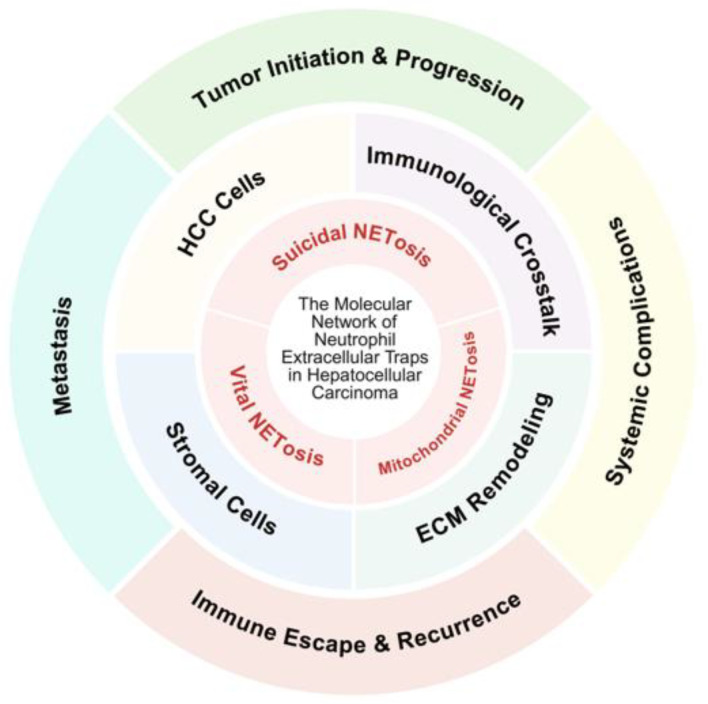
Conceptual framework of the multifaceted roles of NETs in the HCC ecosystem.

In this review, we systematically define the molecular regulatory network of NETs in HCC. We outline the mechanisms governing NETs formation and clarify their multidimensional roles in HCC initiation, metastasis, and drug resistance. We also assess the translational potential of NETs as diagnostic biomarkers and therapeutic targets. By integrating current evidence on the interaction between NETs and the HCC microenvironment, we aim to provide a clear theoretical foundation for future mechanistic studies and precision therapeutic strategies (Figure 1).

## 2. The Molecular Mechanisms of NETs Formation

**Figure 2:**
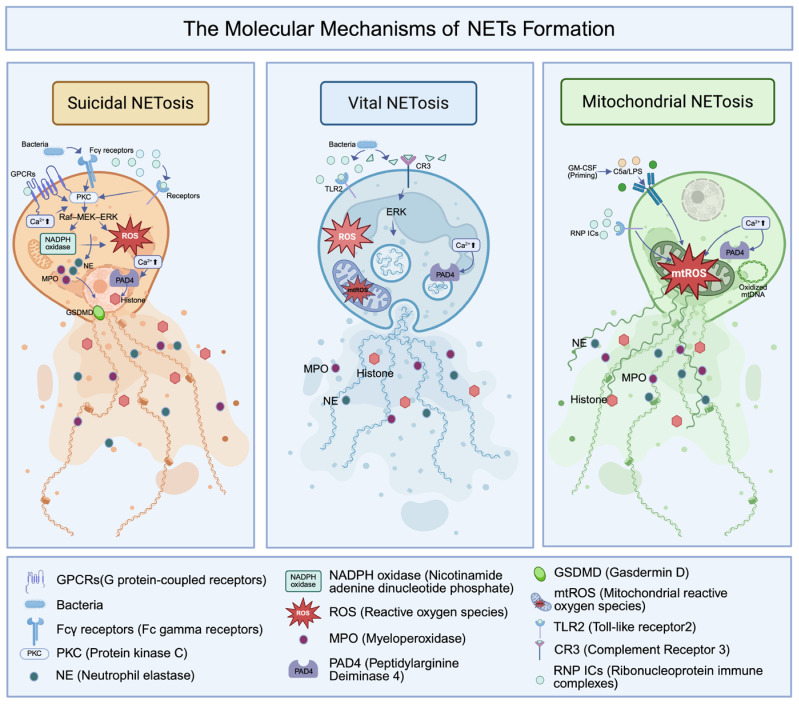
Molecular pathways of NETs formation through different NETosis modes.

NETs were first described in 2004 as extracellular, web-like structures composed of decondensed chromatin and cytotoxic granule proteins [10]. In 2007, researchers formally defined the underlying cell death program as “NETosis”, which differs from classical apoptosis and necrosis [11,28]. Over the past two decades, the conceptual framework of NETosis has evolved from a single pathway to a complex network of distinct mechanisms. Current consensus classifies NETs formation into three main types. Suicidal NETosis represents the classical lytic form, characterized by plasma membrane rupture and release of nuclear DNA [28]. Vital NETosis occurs rapidly and does not cause cell lysis; neutrophils remain viable and retain effector functions after releasing chromatin [29,30,31]. Mitochondrial NETosis involves the selective release of mitochondrial DNA (mtDNA) rather than nuclear chromatin [32]. In this section, we summarize the major triggers, morphological features, and key molecular signaling pathways of these three NETosis phenotypes (Figure 2).

### 2.1. Suicidal NETosis: The NADPH Oxidase-Dependent Pathway

Suicidal NETosis is the most thoroughly characterized form of NETs formation. It represents an irreversible, lytic cell death program that typically unfolds over 2–4 h and culminates in plasma membrane rupture with the release of nuclear chromatin [28,29]. Neutrophils initiate suicidal NETosis when they detect exogenous or endogenous danger signals through surface receptors, including G protein-coupled receptors (GPCRs) and Fcγ receptors [33,34]. Classical stimuli include phorbol 12-myristate 13-acetate (PMA), bacteria, fungi, cholesterol crystals, and immune complexes [28,35]. Upon receptor engagement, protein kinase C (PKC) activation is initiated, further triggering the Raf–MEK–ERK signaling pathway. This cascade reaction leads to phosphorylation of the cytosolic subunit p47phox, promoting the assembly of the functional NADPH oxidase 2 (NOX2) complex on the plasma membrane [36,37]. In parallel, stimuli such as PMA induce a sustained rise in intracellular Ca^2+^ levels. This calcium influx synergizes with PKC isoforms to facilitate NOX2 assembly and creates the ionic conditions required to activate peptidylarginine deiminase 4 (PAD4) [38,39,40]. The assembled NOX2 complex then generates reactive oxygen species (ROS), which act as essential upstream signaling mediators in suicidal NETosis [28].

After ROS production, NE is released from azurophilic granules into the cytosol and then translocates to the nucleus. In the nucleus, NE cleaves core histones, particularly H4, thereby initiating chromatin relaxation. MPO subsequently enters the nucleus, where its strong cationic properties enable it to bind chromatin and cooperate with NE to promote chromatin decondensation. Neutrophils from MPO-deficient patients or cells treated with MPO inhibitors show markedly reduced NETs formation [41]. As chromatin remodeling progresses, higher-order chromatin structures collapse, allowing decondensed chromatin to mix with cytoplasmic granule proteins [42]. At the same time, PAD4 catalyzes extensive histone citrullination by converting positively charged arginine residues into neutral citrulline. This modification weakens histone–DNA interactions and drives chromatin swelling [40]. Genetic ablation of PAD4 completely blocks NETs formation induced by diverse physiological stimuli, underscoring its central role [43].

Recent studies further identify the pore-forming protein gasdermin D (GSDMD) as a critical mediator of the terminal stage of NETosis. After cleavage by inflammatory caspases, activated GSDMD forms pores in the nuclear envelope and plasma membrane. These pores facilitate nuclear–cytoplasmic mixing and accelerate extracellular release of decondensed chromatin. Accordingly, genetic or functional deficiency of GSDMD markedly impairs NETs release [44]. Ultimately, the nuclear envelope disintegrates, and the plasma membrane ruptures, releasing the antimicrobial chromatin network into the extracellular space and completing suicidal NETosis [28,45].

### 2.2. Vital NETosis: Rapid Release Without Cell Death

In contrast to the slower, lytic process of suicidal NETosis, vital NETosis is rapid and non-lytic. Neutrophils undergoing vital NETosis release NETs while preserving full viability and functional capacity. This response typically occurs within 5–60 min after stimulation, allowing NET release to coexist with ongoing host defense functions [29,30,46]. Importantly, vital NETosis often proceeds independently of NADPH oxidase–derived ROS [47].

Pathogen recognition and microenvironmental cues tightly regulate vital NETosis. Bacterial components activate this pathway through Toll-like receptor 2 (TLR2) and complement receptor 3 (CR3) [31]. Intercellular interactions further amplify this response. Platelets detect bacterial signals through Toll-like receptor 4 (TLR4), become activated, and directly interact with neutrophils to trigger rapid NET release. The resulting NETs remain stable under intravascular shear stress [46,48]. The extracellular matrix (ECM) also contributes to regulation. For example, fibronectin cooperates with fungal β-glucan to activate vital NETosis through the CR3–ERK signaling axis [47].

Vital NETosis displays distinct morphological features, especially in nuclear dynamics. After stimulation, the nucleus rapidly rounds and condenses. The nuclear envelope then undergoes vesiculation, forming DNA-containing vesicles that traffic through the cytoplasm and fuse with the intact plasma membrane. This exocytic mechanism releases extracellular chromatin without disrupting membrane integrity or causing cell lysis [30]. The resulting anuclear polymorphonuclear cells (PMNs) retain intact granules and plasma membranes. Intravital imaging studies show that these anuclear PMNs preserve key functions, including chemotaxis and phagocytosis [31].

### 2.3. Mitochondrial NETosis: ROS-Dependent Extrusion of mtDNA

The third distinct form of NETs formation is mitochondrial NETosis, which involves the selective release of mtDNA rather than nuclear chromatin. Unlike the lytic suicidal pathway, mitochondrial NETosis does not cause cell death or plasma membrane rupture. It typically occurs rapidly, within approximately 15 min. Complement factor 5a (C5a) or lipopolysaccharide (LPS) can trigger this response, particularly after priming with granulocyte/macrophage colony-stimulating factor (GM-CSF) [32].

Mitochondrial reactive oxygen species (mtROS) act as the central initiators of mitochondrial NETosis. Ribonucleoprotein immune complexes (RNP ICs) induce mitochondrial depolarization and robust mtROS production, which then drives NET release. This mechanism operates independently of NOX, as demonstrated in patients with chronic granulomatous disease (CGD). Although their neutrophils lack functional NOX2, they still generate mitochondrial NETs [49,50]. Specific mtROS scavengers, such as MitoTEMPO, completely block this process [50]. In addition, calcium (Ca^2+^) influx serves as a critical trigger in this NOX-independent pathway. Calcium ionophores, including A23187, induce mitochondrial NETosis by activating the calcium-activated small conductance potassium channel 3 (SK3) [51]. Concurrently, Ca^2+^ influx activates PAD4, promoting chromatin decondensation and NETs release [52]. Beyond classical immune stimuli, bacterial metabolites also regulate mitochondrial NETosis. Certain pathogens, such as *Staphylococcus aureus*, produce lactic acid within phagosomes. Lactic acid enters neutrophil mitochondria and disrupts the electron transport chain, thereby accelerating mtROS production and initiating NETosis. This mechanism directly links microbial metabolic activity to host innate immune responses [53].

Notably, the mtDNA released during mitochondrial NETosis serves a dual role. It not only constitutes a structural component of NETs but also acts as a potent proinflammatory signaling molecule. Oxidized mtDNA can be internalized by recipient cells, where it activates the cyclic GMP-AMP synthase (cGAS)–STING pathway and induces the production of type I interferons and multiple proinflammatory cytokines [50,54]. In turn, activation of the mtDNA–cGAS–STING axis further amplifies NETs release, establishing a feed-forward inflammatory loop. This signaling cascade contributes to the pathogenesis of inflammatory diseases, tissue injury, and tumor-associated microenvironments [54].

## 3. The Origins of NETs in the HCC Microenvironment

**Figure 3:**
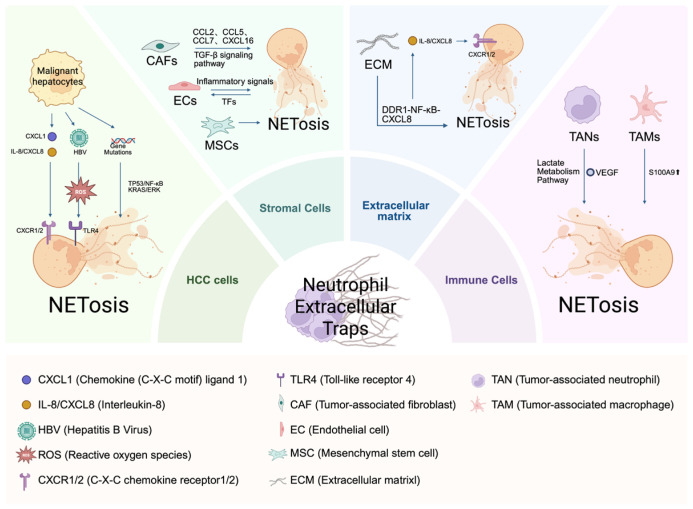
Multidimensional tumor microenvironment network triggering NETosis in HCC.

NETs were initially described as innate immune structures that eliminate invading pathogens. However, their functional scope now extends beyond infection to sterile inflammation, particularly in cancer [37]. HCC develops within a highly complex TME composed of cellular and non-cellular components. The cellular compartment includes malignant hepatocytes as well as non-malignant populations, such as immune cells and stromal cells, embedded within an extracellular matrix framework [55,56]. This TME sustains chronic inflammation, hypoxia, and immunosuppression, all of which strongly activate infiltrating neutrophils [57]. As a result, the HCC microenvironment contains persistent stimulatory signals that continuously drive NETosis. In this section, we examine the principal cellular sources and molecular mediators that initiate and sustain NETs formation within the HCC ecosystem (Figure 3).

### 3.1. HCC Cells Trigger NETosis

Malignant hepatocytes serve as primary drivers of NETosis within the HCC tumor microenvironment. They activate neutrophils by releasing a secretome enriched in proinflammatory cytokines and chemokines [9]. Interleukin-8 (IL-8/CXCL8) represents a central mediator of this crosstalk. Clinical studies show that intratumoral IL-8 levels positively correlate with NETs density [58,59]. Mechanistically, HCC-derived IL-8 binds to C-X-C chemokine receptors 1 and 2 (CXCR1/2) on neutrophils, promoting assembly of the NOX2 complex and triggering ROS-dependent suicidal NETosis [58,60]. The capacity of HCC cells to induce NETs also reflects their metabolic and genetic context. At the epigenetic level, acetyl-CoA accumulation—driven by downregulation of acetyl-CoA thioesterase 12 (ACOT12) and upregulation of acyl-CoA synthetase short-chain family members 1 and 2 (ACSS1/2)—enhances CXCL1 expression. This shift establishes the CXCL1–CXCR2 signaling axis, recruits neutrophils, and promotes NETs formation [61].

Oncogenic mutations further amplify NETosis. TP53 mutations sustain high IL-8 secretion through constitutive activation of NF-κB signaling [62,63]. KRAS signaling enhances NETs formation via the Ras/Raf/MEK/ERK pathway [36,64,65,66]. Moreover, elevated NETs risk scores associate with CTNNB1 and MUC16 mutations, indicating that genetic alterations influence neutrophil activation and NETs propensity [62].

Viral etiology adds another layer of regulation. HBV-positive HCC cells induce NETs formation more effectively than non-tumor hepatocytes. This effect depends on the damage-associated molecular pattern (DAMP) protein S100A9. Silencing S100A9 markedly reduces NETs formation, suggesting that virus–tumor interactions further amplify NETosis within the HCC microenvironment [15].

The HCC microenvironment also induces mitochondrial NETosis. HCC-derived factors increase mtROS in neutrophils, triggering the release of NETs enriched in ox-mtDNA. HCC cells internalize this ox-mtDNA, which upregulates key inflammatory mediators, including cyclooxygenase-2 (COX-2), IL-8, and IL-6. This interaction establishes a malignant positive feedback loop that sustains NET formation, amplifies tumor-associated inflammation, and accelerates disease progression [20,67].

### 3.2. Stromal Cells in the HCC Microenvironment Trigger NETosis

The HCC TME contains diverse stromal cell populations, including hepatic stellate cells (HSCs), cancer-associated fibroblasts (CAFs), tumor endothelial cells (TECs), and mesenchymal stem cells (MSCs). These stromal cells drive tumor progression by promoting metabolic reprogramming, angiogenesis, and immune evasion, while simultaneously creating conditions that favor NETs formation [68,69,70].

Activated HSCs and CAFs serve as key inducers of NETosis within the TME. CAFs in HCC markedly increase secretion of chemokines such as CCL2, CCL5, CCL7, and CXCL16. These chemokines recruit neutrophils and enhance HCC cell migration and invasion through activation of Hedgehog and TGF-β signaling pathways [71,72]. Among these mediators, TGF-β1 acts as a central driver of NETosis. In HCC patient tissues, TGF-β1 expression positively correlates with levels of MPO–DNA complexes. Both in vitro and in vivo experiments demonstrate that TGF-β1 stimulates neutrophils to release CitH3-enriched NETs, and TGF-β neutralizing antibodies significantly inhibit this effect [18]. NETs, in turn, regulate HSC function. NETs activate the TLR3/COX-2 pathway in HSCs, enhancing mitochondrial respiration and glycolysis. This metabolic reprogramming promotes HSC proliferation and differentiation into myofibroblasts. As a result, NETs and activated HSCs form a reinforcing positive feedback loop within the HCC microenvironment [73].

Crosstalk between NETs and the vascular system further shapes the inflammatory microenvironment and provides a mechanistic basis for immune thrombosis. Although direct evidence in HCC remains limited, thrombo-inflammatory models demonstrate a strong interaction between NETs and endothelial cells (ECs). Endothelial cells internalize NETs, which alters inflammatory signaling pathways [74]. NETs also disrupt intercellular junctions between ECs and upregulate tissue factor (TF) expression, thereby driving a procoagulant endothelial phenotype. This process intensifies local inflammation and promotes cancer-associated venous thrombosis [75,76]. The NETs–endothelial activation–thrombosis axis likely contributes to vascular remodeling and thrombo-inflammation within the HCC microenvironment.

MSCs regulate NETosis in a context-dependent manner. In non-tumor settings, MSCs suppress inflammation by reducing PAD4 expression and ROS production, thereby limiting NETs formation [77,78,79]. In contrast, within the tumor microenvironment, MSCs frequently undergo functional reprogramming. For example, gastric cancer-derived MSCs prolong neutrophil survival and enhance their activation through the IL-6–STAT3–ERK1/2 signaling pathway [80]. TNF-α–primed MSCs further polarize neutrophils toward an immunosuppressive phenotype resembling granulocytic myeloid-derived suppressor cells (G-MDSCs) [81]. These findings highlight the pronounced functional plasticity of MSCs in modulating NET-associated networks within the tumor microenvironment.

### 3.3. Extracellular Matrix (ECM) in the HCC Microenvironment Trigger NETosis

The ECM within the TME functions not only as a structural scaffold but also as a dynamic reservoir of cytokines and growth factors that regulate cancer and immune cell behavior [82]. In HCC, chronic inflammation and fibrosis drive extensive ECM remodeling. This remodeling increases ECM mass and stiffness and enriches the matrix with collagen, particularly type I collagen (Col1). These structural alterations activate signaling pathways, including integrin-mediated cascades, which promote angiogenesis, sustain chronic inflammation, and reinforce immunosuppression, thereby accelerating tumor progression [83].

Recent multi-omics analyses demonstrate a strong positive correlation among high ECM stiffness scores, Col1 deposition, and neutrophil infiltration. Importantly, increased neutrophil accumulation coincides with functional exhaustion of CD^8+^ T-cells. Mechanistically, Col1 acts as a central mediator. Col1 binds to discoidin domain receptor 1 (DDR1) on HCC cells and activates the NF-κB signaling axis. This activation upregulates CXCL8 (IL-8) secretion, which recruits neutrophils and induces NETosis. The resulting ECM-induced NETs form a physical barrier around tumor clusters. This barrier spatially restricts CD^8+^ T-cell infiltration and limits direct cytotoxic contact, thereby reinforcing an immune-excluded phenotype [84].

NET-associated histones further activate hepatic stellate cells through the TLR4–MyD88 pathway. This activation enhances collagen deposition, drives additional ECM remodeling, and exacerbates progressive fibrosis, which sustains pro-NETosis signaling [85]. Thus, ECM remodeling in HCC acts both as a driver and a consequence of NETs formation. Together, ECM alterations and NETs establish a fibrotic, immunosuppressive tumor microenvironment that supports HCC progression.

### 3.4. Immunological Crosstalk: Immune Cells and NETs in HCC

Although certain immune cells preserve antitumor activity by clearing NETs, accumulated NETs frequently drive immune cell dysfunction within the tumor microenvironment [59,86,87,88]. In the HCC TME, dysregulated crosstalk between neutrophils and other immune populations promotes pathological NET accumulation and deepens immunosuppression.

TANs display marked functional plasticity. Among these subsets, the N2 phenotype acts as a major driver of tumor progression and NET formation [89]. A high neutrophil-to-lymphocyte ratio (NLR) correlates with increased secretion of proinflammatory cytokines and enrichment of N2-type TANs. These N2 neutrophils release NETs that induce functional exhaustion of adjacent CD^8+^ T-cells, thereby contributing to poor clinical outcomes [90]. Metabolic reprogramming further reinforces this phenotype. Lactic acid, which accumulates in malignant ascites, functions as a signaling molecule that upregulates PD-L1 expression on neutrophils and promotes their protumor polarization and NETs release through the VEGF signaling axis [91].

Tumor-associated macrophages (TAMs), particularly the M2 subtype, represent another key inducer of NETosis in the TME. The M2d subset forms a positive feedback loop with neutrophils through the NETs–IL-17/VEGF/S100A9 signaling axis. NETs-derived IL-17 promotes M2d polarization, and polarized M2d macrophages further enhance NETosis by increasing S100A9 expression, thereby accelerating extrahepatic metastasis [92]. In addition, TAM-derived extracellular vesicles (EVs) carrying fibrinogen-like 2 (FGL2) bind to the FCGR2B receptor on tumor cells. This interaction enhances cancer stemness and indirectly stimulates neutrophils to release NETs, establishing an immunosuppressive barrier at invasive fronts [93]. At the metabolic level, CD39 expressed on macrophages and CD73 expressed on tumor cells cooperatively generate adenosine. Adenosine activates the adenosine receptor 2A (A2AR), upregulates CXCL5 secretion, and creates a chemotactic gradient that recruits neutrophils and triggers NETosis [94].

## 4. The Roles of NETs in HCC Progression

**Figure 4:**
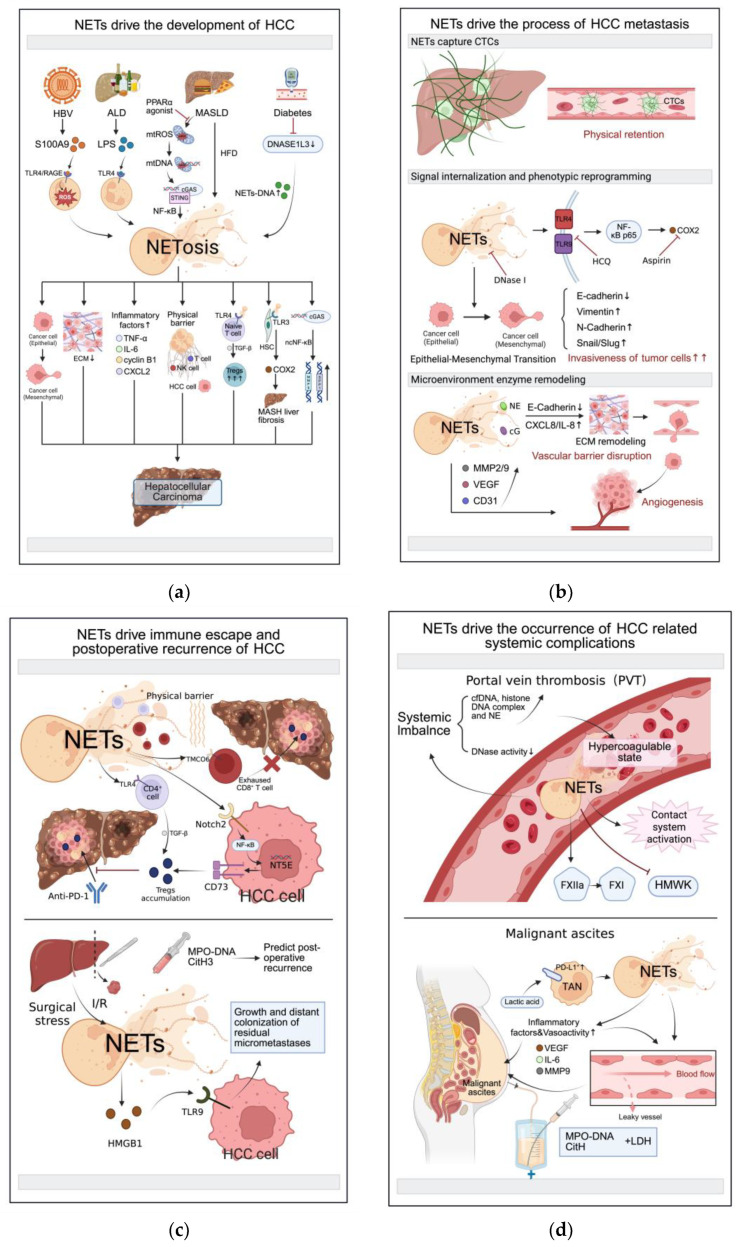
Multifaceted roles of NETs in hepatocellular carcinoma. (**a**) NETs drive initiation and progression of HCC. (**b**) NETs promote HCC metastasis by capturing circulating tumor cells and inducing epithelial–mesenchymal transition. (**c**) NETs facilitate immune evasion and postoperative recurrence of HCC via physical and molecular barriers. (**d**) NETs contribute to HCC-associated systemic complications, including portal vein thrombosis and malignant ascites.

NETs critically drive the progression of HCC. As key immune effectors associated with chronic inflammation, NETs actively reshape the TME and promote tumor initiation, immune evasion, metastasis, and recurrence. Through these mechanisms, NETs function as central mediators of malignant progression and emerge as promising diagnostic biomarkers and therapeutic targets. Although the complete molecular network that governs NETs activity in HCC remains incompletely defined, current evidence supports a multifaceted role in disease evolution. In this section, we systematically examine how NETs contribute to tumorigenesis, facilitate metastasis, mediate therapeutic resistance, and promote HCC-related complications (Table 1 and Figure 4).

### 4.1. NETs Drive the Development of HCC

Chronic inflammation forms the central pathological foundation of HCC. Although vaccination and antiviral therapies have reduced the incidence of virus-related HCC, non-viral etiologies—including metabolic dysfunction-associated steatotic liver disease (MASLD), alcoholic liver disease (ALD), and diabetes—now drive a growing proportion of HCC cases worldwide [110]. Across these distinct disease settings, NETs function as critical molecular hubs that connect metabolic dysregulation and sterile inflammation to malignant transformation.

In HBV-associated HCC, viral infection initiates hepatocarcinogenesis by stimulating hepatocytes to release DAMPs, particularly S100A9. S100A9 binds to TLR4 and receptor for advanced glycation end products (RAGE) on neutrophils, activates downstream ROS signaling, and robustly induces NETosis. The resulting NETs promote tumor progression by driving epithelial–mesenchymal transition (EMT) and degrading the ECM [15].

Differing from viral mechanisms, the pathogenesis of alcoholic liver disease (ALD) is fundamentally regulated by the gut–liver axis. Chronic ethanol consumption disrupts the intestinal barrier and facilitates translocation of bacterial LPS into the liver. As a key pathogen-associated molecular pattern (PAMP), LPS activates TLR4 on neutrophils and triggers extensive NET release, thereby aggravating hepatic inflammation and injury. Experimental antibiotic treatment or genetic deletion of NE and TLR4 significantly reduces alcohol-induced steatosis and hepatocarcinogenesis. These findings establish the gut–LPS–TLR4–NETs axis as a central driver in ALD-associated HCC [95].

In MASLD, lipid metabolic dysfunction and high-fat diets strongly induce NETs formation [67,96]. These NETs exert potent oncogenic effects through several mechanisms. First, they create a physical barrier that excludes cytotoxic CD^8+^ T-cells and natural killer (NK) cells, thereby establishing an immune-cold microenvironment. Second, they reprogram naïve CD^4+^ T-cells into immunosuppressive Tregs by enhancing oxidative phosphorylation [67,102]. In addition, NETs activate HSCs through the TLR3/COX-2/prostaglandin E2 (PGE2) signaling pathway, promoting fibrosis in MASLD and creating a permissive niche for hepatocarcinogenesis [73].

Diabetes further amplifies NET formation in a hyperglycemic milieu. Mechanistically, reduced expression of the extracellular DNA-degrading enzyme DNASE1L3 in HCC leads to accumulation of NET-derived DNA within the TME. HCC cells recognize this persistent extracellular DNA through cyclic cGAS, which activates non-canonical NF-κB signaling. This pathway upregulates invasion-associated genes, including MMP9 and SPP1, thereby enhancing malignant invasion [111].

Collectively, regardless of etiology, NETs facilitate the transition from chronic liver disease to malignancy and drive subsequent tumor progression and invasion by sustaining inflammation and weakening antitumor immunity.

### 4.2. NETs Drive the Process of HCC Metastasis

NETs function as multifaceted drivers of HCC metastasis. They promote metastatic spread through three principal mechanisms: physical trapping of tumor cells, activation of intracellular signaling pathways, and remodeling of the tumor microenvironment.

The most direct pro-metastatic effect of NETs arises from their web-like DNA structure, which serves as a physical scaffold within the circulation. These extracellular DNA networks mechanically capture and anchor circulating tumor cells (CTCs), thereby increasing their retention and colonization within hepatic and pulmonary capillary beds [15,16]. This adhesive interaction protects CTCs from hemodynamic shear stress and facilitates early implantation at distant sites, providing a structural foundation for micrometastasis formation [15,98].

Beyond mechanical trapping, NETs actively reprogram tumor cell behavior. Captured HCC cells internalize NET components, which function as signaling ligands that alter tumor cell phenotype. Internalized NETs activate the TLR4/9–COX2 signaling axis, upregulate inflammatory mediators such as COX2 and IL-8, and enhance metastatic capacity [16]. Critically, NETs induce EMT, marked by downregulation of the epithelial marker E-cadherin and upregulation of mesenchymal markers, including vimentin and N-cadherin, as well as EMT-associated transcription factors such as Snail and Slug. This phenotypic shift markedly increases cellular motility and invasiveness [15,60,99,100]. Moreover, NETs enriched in ox-mtDNA exert stronger proinflammatory effects than those containing nuclear DNA, enabling more potent activation of prometastatic signaling pathways [20].

Concurrently, NETs actively remodel metastatic niches through proteases embedded within their structure, thereby facilitating tumor cell extravasation and outgrowth. NE and cathepsin G associated with NETs directly degrade components of the ECM and disrupt endothelial tight junction proteins. This proteolytic activity increases vascular permeability and enables tumor cells to penetrate the endothelial barrier more efficiently [60,99,100]. After successful colonization, NETs further drive the transition from micrometastases to macrometastatic lesions by inducing overexpression of vascular endothelial growth factor (VEGF) and matrix metalloproteinase 9 (MMP9). These factors promote endothelial lumen formation and neovascularization, thereby securing an adequate blood supply to sustain metastatic expansion [15].

### 4.3. NETs Drive Immune Escape and Postoperative Recurrence of HCC

A defining feature of HCC progression is the development of immune escape. Tumor cells evade immune surveillance through multiple mechanisms, allowing uncontrolled growth and dissemination [112]. Immune escape not only underlies poor responses to therapy and resistance to ICIs but also contributes substantially to the high rate of postoperative recurrence [5]. Emerging evidence identifies NETs as critical regulators within the immune escape network of HCC.

NETs promote the accumulation of immunosuppressive cells and weaken antitumor immunity by reshaping the TME. Clinical analyses show that a high NETs score positively correlates with the tumor immune dysfunction and exclusion (TIDE) score in patients with HCC, supporting a direct role for NETs in immune resistance. Mechanistically, NET-derived DNA activates the Notch2–NF-κB signaling axis in tumor cells, upregulates CD73 expression, and enhances Treg infiltration. This pathway amplifies adenosine-mediated immunosuppression within the TME [101]. In MASLD-associated HCC, NETs also reprogram naïve CD^4+^ T-cells through TLR4 signaling, driving their differentiation into Tregs and reinforcing immune tolerance [102]. Beyond recruiting suppressive immune populations, NETs directly impair cytotoxic T lymphocyte (CTL) function. NET-DNA binds to TMCO6 on CD^8+^ T-cells, suppresses T-cell receptor (TCR) signaling, and blocks nuclear translocation of NF-κB p63. These effects induce T-cell apoptosis and functional exhaustion [18]. In Col1-rich cirrhotic stroma, NETs generated through the Col1–DDR1–CXCL8 axis form a physical barrier around tumor clusters, limiting T-cell infiltration and reducing the efficacy of ICIs [84]. Additionally, activation of the GSK3A–LRG1 axis in tumor cells promotes neutrophil chemotaxis and NETs formation, which further suppresses cytotoxic T-cell activity and facilitates immune escape [103].

NETs play a pivotal role in postoperative recurrence of HCC. Surgical stress and hepatic ischemia–reperfusion (I/R) injury trigger a rapid and robust release of NETs from activated neutrophils. These NETs promote the proliferation and distant seeding of residual micrometastases by releasing high-mobility group box 1 (HMGB1) and activating the TLR9 signaling pathway in tumor cells [104]. Clinical studies demonstrate that patients with elevated preoperative serum MPO–DNA complexes or high intratumoral citrullinated histone H3 (Cit-H3) levels experience significantly shorter recurrence-free survival (RFS) and overall survival (OS). These data identify NETs as independent prognostic indicators of postoperative recurrence [105]. Based on these mechanistic insights, researchers have developed injectable hydrogels and self-gelling powders containing DNase I to locally degrade NETs at surgical margins. This strategy removes the NETs-derived physical barrier and alleviates NK cell suppression, thereby significantly lowering the risk of postoperative recurrence [106,107].

### 4.4. NETs Drive the Occurrence of HCC Related Systemic Complications

The clinical course of HCC is often complicated by severe systemic events, including portal vein thrombosis (PVT) and malignant ascites [113]. Increasing evidence identifies NETs as central molecular mediators in these processes, linking tumor progression with systemic vascular dysfunction and inflammatory dysregulation.

In PVT, which can progress to liver failure and portal hypertension, NETs establish a prothrombotic state known as immune thrombosis. Studies in patients with decompensated cirrhosis and HCC reveal a systemic imbalance in NETs homeostasis, characterized by excessive NETs production—reflected by elevated levels of cell-free DNA (cfDNA), histone–DNA complexes, and NE—and impaired degradation due to reduced DNase activity. This imbalance directly promotes a hypercoagulable state [114]. NETs further drive thrombosis by activating the contact coagulation system. Their negatively charged components provide a surface that activates coagulation factor XII (FXII to FXIIa) and consumes high-molecular-weight kininogen (HMWK). The extent of this activation correlates positively with disease severity, as measured by the model for end-stage liver disease (MELD) score, and independently predicts thrombotic events in patients with HCC [108].

NETs also play a central role in the development of malignant ascites. Compared with peripheral blood, ascitic fluid from patients with HCC contains significantly higher levels of NET markers, including MPO–DNA complexes and CitH3. When combined with conventional biomarkers such as lactate dehydrogenase (LDH), these NET-associated markers markedly improve diagnostic accuracy. A metabolic–inflammatory feedback loop sustains this pathological fluid microenvironment. High concentrations of lactic acid in the peritoneal cavity upregulate PD-L1 expression on neutrophils and trigger NET release [91]. In turn, NETs enhance vascular activity and stimulate secretion of inflammatory mediators, including IL-6, VEGF, and MMP-9. These changes increase vascular permeability and promote fluid accumulation, thereby driving ascites formation [109].

## 5. Clinical Translation: NETs as Biomarkers and Therapeutic Targets

**Figure 5:**
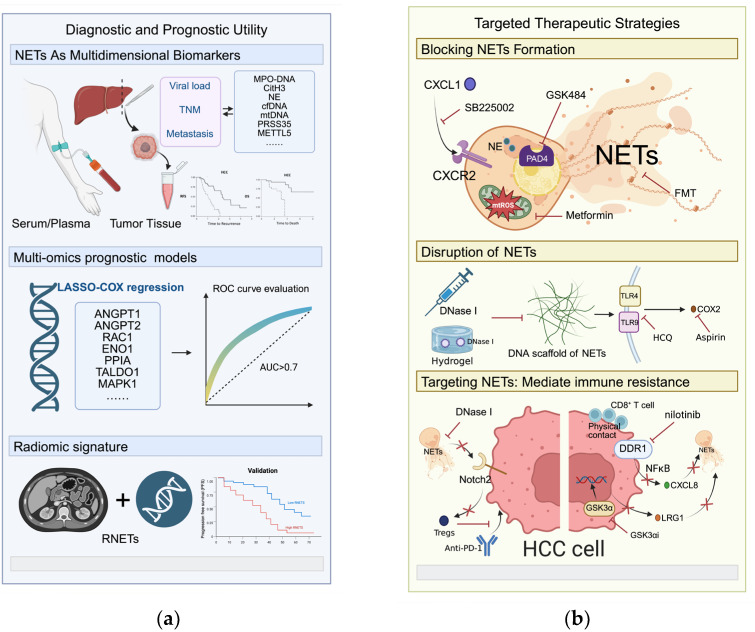
Diagnostic, prognostic, and therapeutic strategies targeting NETs in HCC. (**a**) Diagnostic and prognostic utility of NETs as multidimensional biomarkers in HCC. (**b**) Targeted therapeutic strategies for modulating NETs and improving immune resistance in HCC.

In recent years, increasing evidence has demonstrated that the components and biogenesis of NETs hold substantial clinical translational value. Abnormal accumulation of NET-associated molecules in blood, ascites, and tumor tissues closely correlates with tumor burden, metastatic potential, immunosuppressive status, and therapeutic response. Moreover, therapeutic strategies targeting NETs show promise in enhancing immunotherapy efficacy and reducing recurrence risk. This section highlights the clinical potential of NETs as diagnostic biomarkers and therapeutic targets in HCC (Figure 5).

### 5.1. Diagnostic and Prognostic Utility: From Liquid Biopsies to Multi-Omics Signatures

NETs hold strong promise as biomarkers for early detection, prognostic stratification, and therapeutic monitoring in HCC (Table 2). Circulating NETs components—particularly cfDNA, MPO–DNA complexes, and CitH3—are significantly elevated in the serum and tumor tissues of patients with HCC. Clinically, high preoperative serum levels of MPO–DNA or CitH3 independently predict shorter RFS and OS [105]. In HBV-related HCC, circulating MPO–DNA levels positively correlate with viral load, TNM stage, and metastatic status. Notably, MPO–DNA outperforms alpha-fetoprotein (AFP) in sensitivity and specificity for predicting extrahepatic metastasis, highlighting its value in monitoring advanced disease [15]. Novel circulating biomarkers, including serum PRSS35 and cell-free mitochondrial DNA (cf-mtDNA), further expand the liquid biopsy repertoire. These markers provide additional insight into tumor microenvironment dynamics and liver injury following transplantation [115,116]. The methyltransferase METTL5 has also emerged as a potential biomarker due to its critical role in NETs formation. METTL5 is upregulated in HCC tissues and correlates strongly with advanced TNM stage and poor clinical outcomes. Mechanistic evidence suggests that METTL5 promotes HCC progression by facilitating NETs induction and release [117].

To improve prognostic accuracy, investigators have developed multiple multi-omics models based on NET-related genes (NRGs). Using approaches such as LASSO-Cox regression, researchers constructed risk scores from different numbers of hub genes (e.g., 34, 11, 6, 5, or 2 genes). These models consistently demonstrate strong predictive performance for overall survival in both training and external validation cohorts, with area under the curve (AUC) values exceeding 0.7 at 1-, 3-, and 5-year time points [62,118,119,120,121]. High-risk scores correlate significantly with advanced pathological stage, higher histological grade, and increased T stage. Importantly, these NET-based risk scores serve as independent prognostic factors in HCC [119,122]. To enhance clinical applicability, several studies have integrated risk scores with clinicopathological variables—such as age, sex, and TNM stage—to construct predictive nomograms. Calibration curves and decision curve analyses confirm the accuracy and clinical utility of these integrated models [119,122,123].

Radiomics provides a complementary, non-invasive strategy to assess the NET-associated tumor microenvironment. Recent studies have established a NET-related radiomic signature (R-NETs) derived from CT imaging features that predicts prognosis and response to anti-PD-1 immunotherapy in patients with HCC. Patients classified into the low R-NETs group show significantly longer overall survival and higher objective response rates than those in the high R-NETs group [23]. This imaging-based approach enables non-invasive risk stratification and supports treatment decision-making using routine radiological data.

**Table 2:** NETs detection: From diagnostic markers to prognostic tools.

Main Detection Category	Purpose of Detection	Detected Component/Method	References
Serum/Plasma Circulating NETs Markers	Prognostic Assessment and Risk Stratification	Preoperative serum MPO–DNA levels correlate with shorter RFS and OS in HCC patients.	[105]
Serum/Plasma Circulating NETs Markers	Prediction of Extrahepatic Metastasis	In HBV-related HCC, circulating NETs levels significantly correlate with and can predict extrahepatic metastasis.	[15]
Tissue NETs Markers	Prognostic Assessment and Risk Stratification	Expression of CitH3 in tumor tissue serves as an independent prognostic factor for postoperative recurrence and OS.	[105,124]
Specific Protein Expression Levels	Key Regulatory Molecules as Markers	Significantly decreased serum levels of the secreted protein PRSS35 in HCC patients are associated with poor prognosis.	[115]
Specific Protein Expression Levels	Key Regulatory Molecules as Markers	High expression of the methyltransferase METTL5 in tumor tissue correlates with adverse prognosis and TNM stage in HCC.	[117]
NET-Related Gene Expression Signatures	Prognostic Assessment and Risk Stratification	Risk scoring models based on transcriptomic data of NET-related genes (NRGs) effectively predict OS in HCC patients and serve as independent prognostic factors.	[119,120,122]
NET-Related Gene Expression Signatures	Prognostic Assessment and Risk Stratification	Prognostic models integrating NETs and m6A methylation-related long non-coding RNAs accurately stratify HCC patients into different risk groups.	[123]
NET-Related Gene Expression Signatures	Prognostic Assessment and Risk Stratification	Prognostic signatures based on NETosis-correlated long non-coding RNAs can distinguish different risk subgroups in HCC.	[121]
Radiomics Biomarkers	Prediction of Immunotherapy Response	CT image-based radiomics features of NETs can predict the response of HCC patients to PD-1 inhibitor immunotherapy (objective response rate).	[23]

### 5.2. Immune-Targeted Therapy: Strategies Against NETs

Given the central role of NETs in HCC initiation, progression, and metastasis, NET-targeted therapy has emerged as a promising strategy (Table 3). One approach focuses on blocking upstream signaling pathways to suppress NET biogenesis at its source. Inhibition of the CXCL1–CXCR2 recruitment axis, for example with SB225002, effectively reduces neutrophil infiltration and NET release driven by elevated acetyl-CoA levels or SPP1 expression [61,125]. Similarly, targeting PAD4 with inhibitors such as GSK484 prevents histone citrullination and directly blocks NETs formation. PAD4 inhibition also sensitizes HCC cells to lenvatinib-induced cuproptosis, thereby overcoming therapeutic resistance [126]. Metabolic reprogramming offers an additional strategy. Metformin inhibits mitochondrial complex I, decreases mtROS production and ox-mtDNA release, and attenuates the pro-metastatic activity of NETs [20]. Fecal microbiota transplantation (FMT) represents a systemic intervention that restores gut microbial balance, reduces excessive neutrophil activation, and suppresses formation of pro-metastatic NETs [127]. Advances in nanotechnology further refine upstream blockade strategies. Peptide-based nanoparticles can selectively bind activated neutrophils and induce in situ assembly of NE–fiber clusters. This approach sequesters NE in the cytoplasm, prevents its nuclear translocation, and inhibits NETosis. At the same time, it forms a physical barrier that limits tumor cell adhesion [100].

In addition to upstream blockade, therapeutic strategies that directly degrade established NETs aim to dismantle their structural scaffold within the TME. DNase I cleaves the extracellular DNA backbone of NETs and has consistently reduced tumor metastasis in multiple preclinical HCC models [60,99]. However, systemic DNase I monotherapy shows limited clinical efficacy because of its short half-life and its inability to remove NET-associated proteins. To overcome these pharmacokinetic constraints, investigators have combined DNase I with anti-inflammatory agents such as aspirin or hydroxychloroquine, which inhibit the TLR4/9–COX2 signaling pathway. This combination strategy produces synergistic antimetastatic effects [16]. For localized control—particularly after surgical resection—advanced delivery systems, including injectable DNase I–loaded hydrogels and self-gelling powders, maintain sustained enzymatic activity at the surgical margin. These approaches remove NET-mediated physical barriers that impair NK cell function and significantly reduce postoperative recurrence [106].

NETs also drive resistance to ICIs, including anti–PD-1 antibodies. By upregulating CD73 expression and promoting Treg infiltration, NETs reinforce an immunosuppressive microenvironment that limits immunotherapy efficacy. Accordingly, degrading NETs with DNase I or inhibiting their formation enhances the antitumor response to PD-1 blockade [101]. Parallel approaches that target the GSK3α–LRG1 axis or inhibit the DDR1 receptor, for example with nilotinib, also reverse neutrophil-mediated immunosuppression. When combined with PD-1 inhibitors, these strategies produce strong synergistic effects. Collectively, these findings indicate that dismantling the NET network is essential for overcoming immune escape and achieving durable clinical responses in HCC [84,103].

## 6. Conclusions and Future Perspectives

As an evolving area of investigation, NETs have become a core and actionable hub in the pathogenesis of HCC. Accumulating evidence demonstrates that NETs are not merely byproducts of inflammation; rather, they actively remodel the TME through their unique three-dimensional DNA scaffold enriched with histones and granule proteins. By amplifying inflammatory cascades, stimulating angiogenesis, shaping a pre-metastatic niche, and suppressing antitumor immune surveillance, NETs drive the transition from chronic liver injury to invasive carcinoma.

However, translating NETs into the framework of precision medicine still faces several critical challenges. Diverse etiological backgrounds, distinct molecular subtypes, and heterogeneous tumor microenvironments likely generate substantial variability in NET-related signaling pathways. Consequently, a comprehensive understanding of the HCC-specific molecular code that triggers and regulates NETs remains elusive. In parallel, the field lacks standardized methods for NET detection and quantification in tissues and body fluids. Studies rely on different biomarkers and cutoff thresholds, which limits cross-study comparability and slows clinical implementation [22]. Furthermore, NETs play an essential physiological role in antimicrobial defense. Therapeutic strategies must therefore selectively target HCC-associated NET activity while preserving systemic immune homeostasis. Achieving this balance represents a central challenge in developing safe and effective NET-directed therapies.

Looking forward, the synergistic integration of multidimensional diagnostic tools is imperative. Combining dynamic liquid biopsies of NET-associated markers with radiomic signatures (R-NETs) and circulating tumor cell analysis offers a promising avenue for the real-time monitoring of the tumor microenvironment. Such an approach could enable the prediction of metastatic risk and the implementation of targeted interventions well before clinical manifestations emerge. On the therapeutic front, future interventions should be tailored to specific pathological drivers. Examples include targeting the gut-LPS in alcohol-related HCC or S100A9 in HBV-associated diseases. Given the role of NETs in constructing physical and immunosuppressive barriers, their targeted elimination should be considered a priming strategy to sensitize immunologically cold tumors to immune checkpoint inhibitors. The shift toward precision is further exemplified by the use of advanced delivery systems for localized, postoperative NETs clearance, as pioneered by DNase I-loaded hydrogels. By systematically elucidating the molecular code of NETs, establishing clinical detection systems, and implementing precise combinatorial intervention strategies, we expect to translate NET-related findings from mechanistic discoveries into clinically valuable biomarkers and therapeutic targets. Ultimately, translating these insights into clinical practice will establish a stronger theoretical foundation and generate innovative treatment strategies to improve the long-term outcomes of patients with HCC.

## Figures and Tables

**Table 1:** The roles of NETs in Hepatocellular Carcinoma.

Mechanism Category	Key Molecules/Pathways	HCC Models Used	Reference
Promoting Tumor Initiation and Development	S100A9/TLR4/RAGE-ROS axis	HBV-stable HCC cell lines (e.g., HepG2.2.15), HBV-related HCC patient samples	[15]
	LPS/TLR4-ROS axis	Ethanol-induced fatty liver and HCC model (ethanol/diethylnitrosamine (DEN) + a 4% Lieber-DeCarli liquid alcohol diet) in mice	[95]
	cGAS-STING-NF-κB-NLRP3-GSDMD axis, ox-mtDNA	HCC patient samples, High-fat diet (HFD) + DEN induced HCC mouse model	[67]
	Free fatty acids direct stimulation	NASH patient samples, STAM mouse model of nonalcoholic steatohepatitis (NASH)-HCC (Streptozotocin + HFD)	[96]
	TLR3/COX-2/PGE2 pathway, HSC activation	Western diet/carbon tetrachloride-induced metabolic dysfunction–associated steatohepatitis (MASH) fibrosis model	[73]
	Metabolic reprogramming, Naive CD^4+^ T-cell entiation into Tregs	STAM mouse model, choline-deficient + HFD + DEN mouse model	[97]
Promoting Tumor Metastasis	VEGF, CD31, EMT markers, MMP2/MMP9	HBV-stable HCC cell lines (e.g., HepG2.2.15), C57BL/6 mice were injected with LPS and HCC cell line H22	[15]
	β2 integrins, ICAM-1, MMP9	Murine Lewis lung carcinoma cell subline H59, A549 human lung carcinoma cell line	[98]
	TLR4/9-COX2, inflammatory response	Immunocompetent C57BL/6 mouse lung metastasis model, Human HCC tissue samples	[16]
	EMT (E-cadherin ↓, Vimentin ↑)	HBV-related HCC cell line (HepG2, HepG2.2.15) and mouse lung metastasis model	[15]
	EMT, E-cadherin ↓, N-Cadherin ↑, Snail/Slug ↑	Human HCC tissue samples, peripheral blood neutrophils from healthy donors + HCC tumor supernatants	[99]
	ox-mtDNA, IL-8, IL-6	Peripheral blood neutrophils from HCC patients, HepG2 cells and mouse lung metastasis model	[20]
	NE, EMT, ECM	The Hepa1-6/luc cell line	[100]
	NET-related cathepsin G (cG), E-cadherin ↓	Neutrophils from HCC patients and healthy donors, Lung metastasis model in nude mice	[60]
Promoting Immune Evasion	NETs → Notch2 → NF-κB → CD73 ↑ → Treg infiltration	Mouse HCC model (hydrodynamic transfection), HCC organoids, clinical HCC samples	[101]
	NETs → TLR4 → CD^4+^ T-cell → Treg → TCR	NASH-HCC mouse models, clinical NASH-HCC patient sample	[102]
	NETs-DNA → TMCO6 (on CD^8+^ T-cells) → Inhibition of TCR/NF-κB → CD^8+^ T cell exhaustion	Wild-type and TMCO6^−/−^ mouse HCC models, clinical HCC samples	[18]
	Liver cirrhosis ECM/Col1 → DDR1 → NF-κB → CXCL8 ↑ → Neutrophil recruitment/NETs barrier	Liver cirrhosis mouse models (DMN-induced), subcutaneous/orthotopic models, humanized mouse models	[84]
	Gsk3a → LRG1 ↑ → Neutrophil recruitment and PD-L1 + NETs → CTL inhibition	C57BL/6 subcutaneous tumor models, immunodeficient NPG mouse models, co-culture systems	[103]
Driving Postoperative Recurrence	Liver ischemia–reperfusion (I/R) → NETs ↑ → HMGB1 release → TLR9 activation	Mouse liver I/R model combined with MC38 colon cancer metastasis model, clinical mCRC patient samples	[104]
	Pre/postoperative serum MPO–DNA, Cit-H3 levels ↑ → Predicts shorter RFS and OS	Cohort of HCC patients undergoing hepatectomy	[105]
	Postoperative local DNase I → Degrades NETs → Breaks physical barrier → Enhances NK/CD^8+^ T-cell immunity → Inhibits recurrence	Mouse liver resection recurrence models, co-culture experiments	[106]
	Radiotherapy (RT) → Induces NETs → Combined with autophagy/CD73/NETs inhibitors → Enhances RT-induced NK cell immunity → Prevents recurrence	Hepa1-6 mouse HCC model	[107]
Promoting Systemic Complications	PVT: NETs markers ↑ (cfDNA, histone-DNA)/DNase activity ↓ → Hypercoagulability	Cohort of decompensated cirrhosis patients (with/without HCC)	[107]
	PVT: NETs → Contact system activation (Factor XIIa ↑, HMWK ↓) → Hypercoagulability	HCC patient cohort (with/without PVT)	[108]
	Malignant Ascites: NETs markers (MPO–DNA, CitH3) ↑ in ascitic fluid vs. benign ascites	HCC patients with malignant ascites vs. benign ascites patient cohort	[91]
	Malignant Ascites: NETs release pro-inflammatory factors (TNF-α, IL-6, IFN-γ), VEGF, MMP-9 → Promotes ascites formation	H22 cell-bearing mouse malignant ascites model	[109]

↑: increase; ↓: decrease; →: promotes.

**Table 3:** Therapeutic strategies targeting NETs.

Therapeutic Mechanism	Target	Compound/Method	References
Inhibition of Neutrophil Chemotaxis and Recruitment	Chemokine receptor CXCR2	SB225002	[61,125]
Inhibition of Key Enzymes for NETs Formation	PAD4	GSK484	[99,126]
Inhibition of Key Enzymes for NETs Formation	NE	Peptide nanomaterial (FTP-NPs)	[100]
Metabolic Modulation to Reduce NETs Generation	Mitochondrial oxidative stress	Metformin	[20]
Metabolic Modulation to Reduce NETs Generation	Peroxisome proliferator-activated receptor alpha (PPARα)	Bezafibrate/Fenofibrate	[67]
Gut Microbiota Modulation to Indirectly Inhibit NETs	Gut microbiota ecology	Fecal microbiota transplantation (FMT) from healthy donors	[127]
Neutralization of NETs-Inducing Factors	Interleukin-33 (IL-33), Mitochondrial complex I subunit NDUFA4L2	IL-33 neutralizing antibody, Gene knockdown (shRNA/siRNA)	[126]
Direct Degradation of NETs Structure	DNA backbone of NETs	Deoxyribonuclease I (DNase I)	[16,20,60,99,101,106,107,125,126]
Blockade of Pro-Metastatic Signaling Upon NETs Internalization	TLR4/9—COX2 signaling pathway	Aspirin/Hydroxychloroquine (HCQ)	[16]
Supplementation/Activation of Endogenous NETs-Inhibitory Proteins	Secreted protease PRSS35	PRSS35 overexpression	[115]
Supplementation/Activation of Endogenous NETs-Inhibitory Proteins	Histidine-rich glycoprotein (HRG)	HRG overexpression	[128]
Postoperative Adjuvant Clearance of Local NETs	NETs and acidic microenvironment at the surgical margin	Injectable hydrogel/powder loaded with DNase I	[106,107]
Sensitization to Immune Checkpoint Inhibitors	Collagen receptor DDR1 (in combination with anti-PD-1)	Nilotinib + Anti-PD-1 antibody	[84]
Sensitization to Immune Checkpoint Inhibitors	NETs-mediated immunosuppressive microenvironment (in combination with anti-PD-1)	DNase I + Anti-PD-1 antibody	[101]
Sensitization to Immune Checkpoint Inhibitors	Glycogen synthase kinase 3 alpha (GSK3a)	SB216763 + Anti-PD-1 antibody	[103]

## Data Availability

No new data were created or analyzed in this study. Data sharing is not applicable to this article.

## Abbreviations

The following abbreviations are used in this manuscript:

HCC	Hepatocellular carcinoma
TME	Tumor microenvironment
NETs	Neutrophil extracellular traps
ICI	Immune checkpoint inhibitor
TAN	Tumor-associated neutrophil
NE	Neutrophil elastase
MPO	Myeloperoxidase
TMCO6	Transmembrane and coiled-coil domains 6
NASH	Non-alcoholic steatohepatitis
Tregs	Regulatory T-cells
Ox-mtDNA	Oxidized mitochondrial DNA
CitH3	Citrullinated histone H3
mtDNA	Mitochondrial DNA
GPCR	G protein-coupled receptor
PMA	Phorbol 12-myristate 13-acetate
PKC	Protein kinase C
NADPH	Nicotinamide adenine dinucleotide phosphate
NOX	NADPH oxidase complex
PAD4	Peptidylarginine Deiminase 4
ROS	Reactive oxygen species
GSDMD	Gasdermin D
TLR	Toll-like receptor
CR3	Complement Receptor 3
ECM	Extracellular matrix
PMN	Polymorphonuclear cell
C5a	Complement factor 5a
LPS	Lipopolysaccharide
GM-CSF	Granulocyte/macrophage colony-stimulating factor
mtROS	Mitochondrial reactive oxygen species
RNP ICs	Ribonucleoprotein immune complexes
CGD	Chronic granulomatous disease
SK3	Small conductance potassium channel 3
cGAS	Cyclic GMP-AMP synthase
IL-8/CXCL8	Interleukin-8
CXCR	C-X-C chemokine receptor
CXCL	Chemokine (C-X-C motif) ligand
ACOT12	Acetyl-CoA thioesterase 12
ACSS1/2	Acyl-CoA synthetase short-chain family member 1/2
HBV	Hepatitis B Virus
DAMP	Damage-associated molecular pattern
COX2	Cyclooxygenase 2
HSC	Hepatic stellate cell
CAF	Tumor-associated fibroblast
TEC	Tumor endothelial cell
MSC	Mesenchymal stem cell
EC	Endothelial cell
TF	Tissue factor
G-MDSC	Granulocyte-myeloid derived suppressor cell
Col1	Type I collagen
DDR1	Discoidin domain receptor 1
NLR	Neutrophil-lymphocyte ratio
TAM	Tumor-associated macrophage
EV	Extracellular vesicle
FGL2	Fibrinogen-like 2
A2AR	Adenosine receptor 2a
DEN	Diethylnitrosamine
HFD	High-fat diet
NASH	Nonalcoholic steatohepatitis
MASH	Metabolic dysfunction–associated steatohepatitis
MASLD	Metabolic dysfunction-associated steatotic liver disease
ALD	Alcoholic liver disease
EMT	Epithelial–mesenchymal transition
PAMP	Pathogen-associated molecular pattern
PGE2	Prostaglandin E2
CTC	Circulating tumor cell
TIDE	Tumor Immune Dysfunction and Exclusion
I/R	Ischemia–reperfusion
RFS	Recurrence-free survival
OS	Overall survival
PVT	Portal vein thrombosis
cfDNA	Cell-free DNA
HMWK	High-molecular-weight kininogen
MELD	Model for end-stage liver disease
LDH	Lactate dehydrogenase
ccf-mtDNA	Circulating free mitochondrial DNA
NRG	NET-related gene
R-NETs	NET-related radiomic signature
FMT	Fecal microbiota transplantation
PPARα	Peroxisome proliferator-activated receptor alpha
DNase I	Deoxyribonuclease I
GSK3a	Glycogen synthase kinase 3 alpha
